# Genetic profiles to identify talents in elite endurance athletes and professional football players

**DOI:** 10.1371/journal.pone.0274880

**Published:** 2022-09-16

**Authors:** David Varillas-Delgado, Esther Morencos, Jorge Gutiérrez-Hellín, Millán Aguilar-Navarro, Alejandro Muñoz, Nuria Mendoza Láiz, Teresa Perucho, Antonio Maestro, Juan José Tellería-Orriols

**Affiliations:** 1 Universidad Francisco de Vitoria, Faculty of Health Sciences, Exercise and Sport Sciences, Pozuelo de Alarcón, Madrid, Spain; 2 VIVO Labs, Alcobendas, Madrid, Spain; 3 Universidad Complutense de Madrid, Faculty of Biological Sciences, Madrid, Spain; 4 Oviedo University, Faculty of Medicine, Hospital Begoña, Gijón, Asturias, Spain; 5 Universidad de Valladolid, Faculty of Medicine, Institute of Biology and Molecular Genetics (IBMG/CSIC), University of Valladolid, Valladolid, Spain; Universiti Malaysia Terengganu, MALAYSIA

## Abstract

The genetic profile that is needed to identify talents has been studied extensively in recent years. The main objective of this investigation was to approach, for the first time, the study of genetic variants in several polygenic profiles and their role in elite endurance and professional football performance by comparing the allelic and genotypic frequencies to the non-athlete population. In this study, genotypic and allelic frequencies were determined in 452 subjects: 292 professional athletes (160 elite endurance athletes and 132 professional football players) and 160 non-athlete subjects. Genotyping of polymorphisms in liver metabolisers (*CYP2D6*, *GSTM1*, *GSTP* and *GSTT*), iron metabolism and energy efficiency (*HFE*, *AMPD1* and *PGC1a*), cardiorespiratory fitness (*ACE*, *NOS3*, *ADRA2A*, *ADRB2* and *BDKRB2*) and muscle injuries (*ACE*, *ACTN3*, *AMPD1*, *CKM* and *MLCK*) was performed by Polymerase Chain Reaction-Single Nucleotide Primer Extension (PCR-SNPE). The combination of the polymorphisms for the “optimal” polygenic profile was quantified using the genotype score (GS) and total genotype score (TGS). Statistical differences were found in the genetic distributions between professional athletes and the non-athlete population in liver metabolism, iron metabolism and energy efficiency, and muscle injuries (p<0.001). The binary logistic regression model showed a favourable OR (odds ratio) of being a professional athlete against a non-athlete in liver metabolism (OR: 1.96; 95% CI: 1.28–3.01; p = 0.002), iron metabolism and energy efficiency (OR: 2.21; 95% CI: 1.42–3.43; p < 0.001), and muscle injuries (OR: 2.70; 95% CI: 1.75–4.16; p < 0.001) in the polymorphisms studied. Genetic distribution in professional athletes as regards endurance (professional cyclists and elite runners) and professional football players shows genetic selection in these sports disciplines.

## Introduction

The role of genetics in athletic performance has been shown in numerous studies to be important in defining the status of both an endurance athlete and a strength athlete [1–4]. Sports performance is a sum of various factors, both extrinsic and intrinsic, that can predict sports performance [5]. At the end of 2020, the total number of deoxyribonucleic acid (DNA) polymorphisms that are significant to athlete status was 97 (35 endurance-related, 24 power-related, and 38 strength-related) [6]. It should be borne in mind however, that hundreds and even thousands of polymorphisms are needed for the prediction of sports performance [7].

Even though numerous studies show an association of genetics with sports performance, there is still a very limited understanding of the role of genetics [8]. Despite this, recent years have witnessed the rise of an emerging market of direct-to-consumer marketing (DTC) tests that claim to be able to identify children’s athletic talents [9, 10]. It is vitally important that sport and exercise medicine practitioners are fully aware of the state of the evidence concerning genetic testing and the limitations of current knowledge [9, 11]. The genetic variants tested most frequently by the companies providing DTC genetic tests related to sport and exercise since 2015 have been those in the α-actinin 3 (*ACTN3*) and angiotensin-converting enzyme (*ACE*) genes, which presumably reflects the fact that more research has been conducted on these polymorphisms than any others in the context of sport and exercise [12–15]. Although the true role of the *ACTN3* c.1729C>T (rs1815739) and *ACE* I/D (rs4340) polymorphisms in skeletal muscle performance and strength traits remains controversial [15–19], in a systematic review the ACE II genotype was associated with physical performance, especially endurance performance, while the ACTN3 CC genotype was associated with speed and power performance [12, 20]. Genetic information may represent a potentially useful tool for current procedures to identify future talent, enhance training or prevent sport-related injuries [7].

For many years, genes with allelic variants have been identified as predisposing individuals to elite performance, including *ACTN3* [19], *ACE* [21], Homeostatic Iron Regulator (*HFE*) [22], Adenosine Monophosphate deaminase 1 (*AMPD1*) [23], among others, but to date, there is limited information on association analysing a larger set of genetic markers regarding sports performance.

Metabolism is key in sports performance because it is ultimately responsible for the ability to perform the necessary skills. Genetics play a relevant role in sports metabolism and several studies have presented the relevance of genes involved in liver metabolism [24, 25], energy efficiency and iron absorption [5] using polygenic profiles as determinants of athletic performance in endurance sports. Previous investigations have found that carrying the T allele in the c.34C>T polymorphism in the *AMPD1* gene (rs17602729) might reduce VO_2_max trainability and lower response to endurance training [23, 26]. The peroxisome proliferator-activated receptor ɤ coactivator 1-α (*PGC1α*) regulates the expression of several genes associated with substrate oxidation, mitochondrial biogenesis, and muscle fibre conversion [27], suggesting that the GG genotype of the c.1444 G>A polymorphism (rs8192678) might facilitate endurance performance [28–30]. Also, genetics play a significant role in interindividual differences in serum iron parameters. The *HFE* gene regulates iron reabsorption [31–33]. Individuals with CG or GG genotypes in the c.187C>G polymorphism (rs1799945) possessed higher circulating iron concentrations inducing a higher haemoglobin concentration [34], associated with greater VO_2_max in professional athletes [35].

The measure of an individual’s peak capacity to perform dynamic aerobic exercise is dependent on the synergistic action of pulmonary, cardiovascular and muscle tissue via a suite of physiological actions that effectively transport and deliver oxygen from the atmosphere to the mitochondria in working muscles [36, 37].

A previous study shows that 125 single nucleotide polymorphisms (SNPs) were analysed regarding cardiorespiratory fitness, but an association with VO_2_max was only found in *ACE* (rs4340), Angiotensin II Receptor Type 1 (*AGTR1*) (rs275652) and Myostatin (*GDF8*) (rs7570532). Ninety-seven genes have been identified as possible predictors of VO_2_max trainability [38]. To verify the strength of these findings and to identify if there are more genetic variants and/or mediators, further tightly controlled studies are required that measure a range of biomarkers across ethnicities [38, 39].

Liver metabolism has recently shown its implication in the performance of elite endurance athletes through several polymorphisms; cytochrome Member 6 of subfamily D of family 2 P450 (*CYP2D6*) c.506-1G>A (rs3892097), isoform 1 of glutathione-S transferase mu (*GSTM1*), glutathione S-transferase pi (*GSTP*) c.313A>G (rs1695) and glutathione S-transferase theta (*GSTT*) implied in detoxify capacity, oxidative stress clearance and recovery of systemic homeostasis in high performance in endurance sports [24].

Several polymorphisms have been associated with muscle injury risk [40]; the creatine kinase isoenzyme MM (*CKM*) gene encodes the cytosolic muscle isoform of creatine kinase responsible for the rapid regeneration of ATP during intensive muscle contraction. The c.*800A>G (rs8111989) polymorphism plays a vital role in the energy homeostasis of muscle cells [41]. The G allele reduce activity of the skeletal muscle in endurance athletes [42, 43]. Also, the c.34C>T polymorphism (rs17602729) of the *AMPD1* gene plays a vital role in the energy metabolism of muscle cells [5]. Subjects with the T allele have diminished exercise capacity and cardiorespiratory responses to exercise in the sedentary state compared to C allele carriers [23, 44]. The TT genotype is associated to metabolic myopathy, with exercise-induced muscle symptoms such as early fatigue, cramps and/or myalgia which are related to the risk of muscle injury [40, 45], especially in football [46]. The *ACTN3* gene has been associated with strength in successful sprinters or track cyclists. The CC genotype is nearly always present among elite power athletes, whereas TT homozygosity, associated with a premature stop codon that produces complete α-actinin-3 deficiency, is more prevalent in some populations of elite endurance athletes, such as marathon runners [47–49]. The TT genotype is associated with a higher incidence and severity of injuries in endurance runners [50, 51] and professional football players [52] with regard to the CC counterparts. Myosin light chain kinase (*MLCK*), a calcium-calmodulin-dependent multi-functional enzyme, plays a critical role in the regulation of smooth muscle contraction [53]. Polymorphisms in the gene that codifies *MLCK* (c.37885C>A (rs28497577) and c.49C>T (rs2700352)) may alter regulatory light chain (RLC) phosphorylation, thus decreasing the ability to produce force and resist tension during voluntary muscle, showing that the C allele of c.37885C>A (rs28497577) polymorphism [53] and T allele of c.49C>T (rs2700352) polymorphism [54] could predispose to higher values of muscle damage during endurance competitions. In the *ACE* gene (rs4340), the deletion (D) allele is habitually more associated with higher activity of the enzyme angiotensin-converting enzyme than the insertion (I) allele [55]. Also, the I allele is associated with the resistance phenotype and the D allele with speed and power phenotypes [19]. Genetic variation in the *ACE* gene might be associated with many heritable traits, including physical, physiological and skill parameters and physical performance [56, 57]. II genotype is associated in the susceptibility to developing muscle injuries among professional football players [58].

The purpose of this study was to compare the differences in liver metabolism, iron metabolism and energy efficiency, cardiorespiratory fitness and muscle injuries polygenic profiles among elite endurance athletes and professional football players with a non-athlete population. In turn, it was intended to demonstrate, in a pioneering way, the genetic differences in these profiles between elite endurance athletes and professional football players.

## Results

All the polymorphisms analysed met the Hardy-Weinberg equilibrium (HWE).

The athletes had a mean age of 24.9 years (±4.9 years): elite endurance athletes 26.1 years (±4.5 years) and professional soccer players 23.4 years (±5.1 years) and the non-athlete population 27.9 years (±4.5 years).

### Polygenic profile of liver metabolism

When adding the genotype scores of *CYP2D6*, *GSTM*, *GSTP* and *GSTT* polymorphisms, the mean value of the TGS in the professional athletes had a value of 69.9 a.u. (±17.3 a.u.), statistical kurtosis: -0.40 (±0.28). The value for the group of elite endurance athletes was 69.7 a.u. (±18.9 a.u.), statistical kurtosis: -0.47 (±0.38) and in professional football players it was 70.2 a.u. (±15.4 a.u.), statistical kurtosis: -0.59 (±0.42). The mean value of the TGS in non-athletes was 63.2 a.u. (±17.8 a.u.) statistical kurtosis: -0.70 (±0.43). The TGS values of the professional athletes and non-athletes were statistically significant (p < 0.001). Similar between elite endurance athletes and professional football players with non-athletes (p = 0.004 and p < 0.001 respectively).

**Fig 1:**
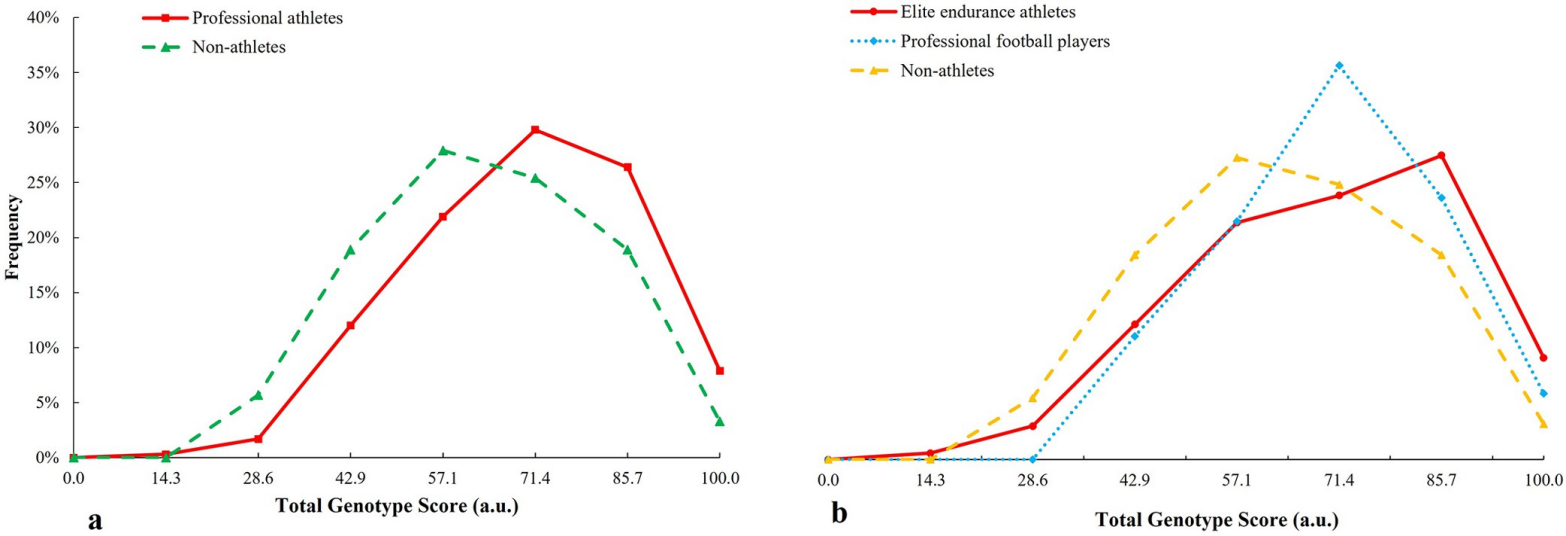
TGS distribution of liver metabolism genes in a) professional athletes and non-athlete subjects and b) elite endurance athletes and professional football players with regard to non-athlete subjects.

TGS distribution of liver-metabolising genes in the professional athletes is shifted to the right with respect to non-athletes (p = 0.022) (Fig 1a), similar between professional football players with non-athletes (p = 0.010) and shows statistical trends with respect to endurance athletes (p = 0.087) (Fig 1b).

**Fig 2:**
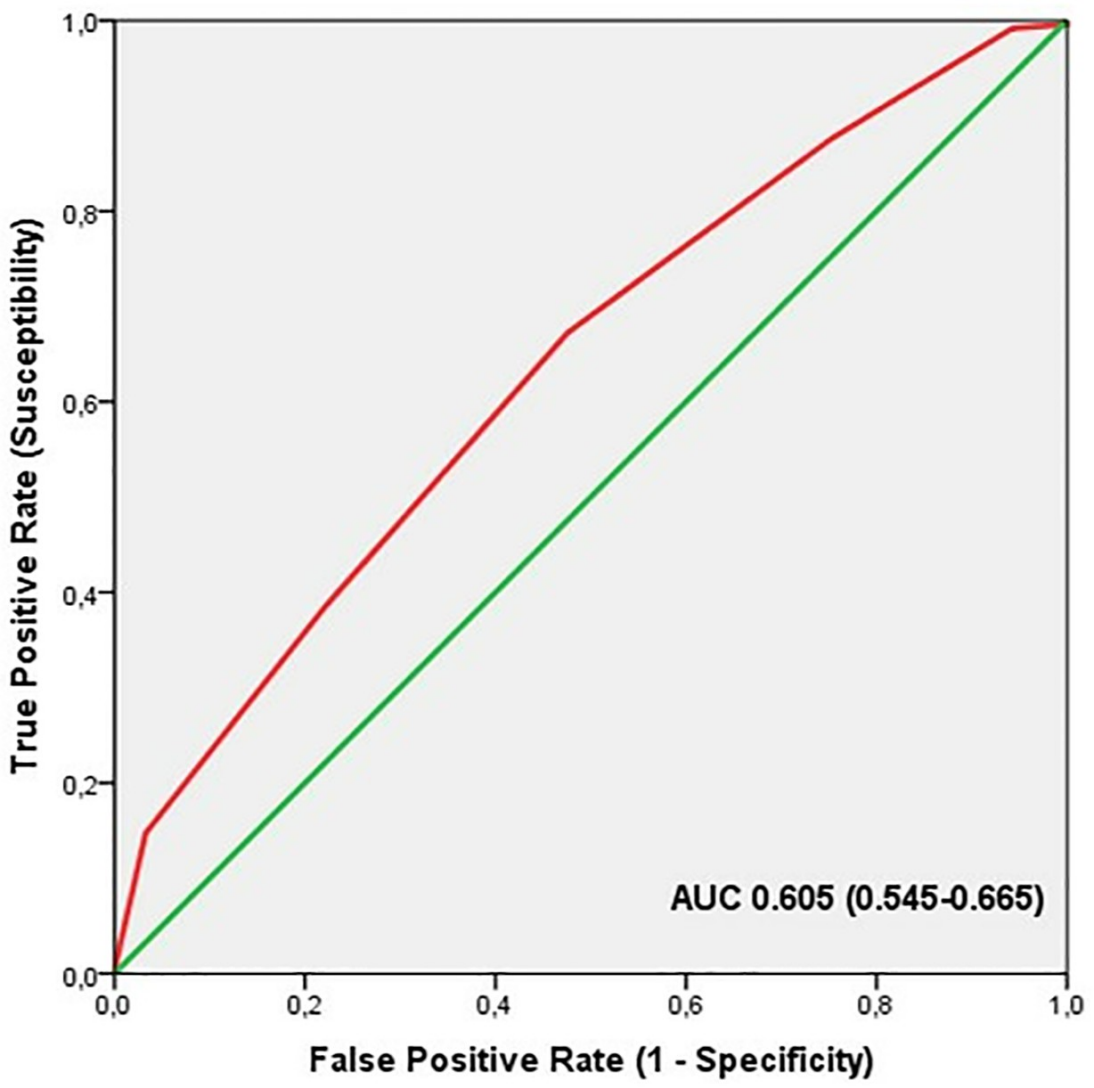
ROC curve summarising the ability of TGS of liver metabolism genes to distinguish potential professional athletes from non-athletes.

ROC analysis showed significant discriminatory accuracy of TGS in the identification of professional athletes (AUC = 0.605; 95% CI: 0.545–0.665; p = 0.001) (sensitivity = 0.640, specificity = 0.475) (Fig 2). The corresponding TGS value at this point was 64.2 a.u. Binary logistic regression analysis showed that subjects with a higher TGS of 64.2 a.u. had an odds ratio (OR) of 1.96 (95% CI: 1.28–3.01; p = 0.002) of being professional athletes, compared to those with a TGS below this value. The elite endurance athletes showed an OR at the cut-off point in comparison to the non-athlete population of 1.79 (95% CI: 1.11–2.88; p = 0.017) and the professional football players, in comparison to non-athlete subjects, had an OR of 2.20 (95% CI: 1.33–3.66; p = 0.001).

Genotype distribution of liver-metabolising genes in the professional athletes’ group, when compared with the non-athlete population, was statistically significant for *CYP2D6* (p < 0.001), showing a higher frequency in the “optimal” genotype in athletes (GG 93.2%) than the non-athlete population (GG 61.1%) (Table 1). Between both groups of professional athletes (endurance and football players), statistically significant results were found in *CYP2D6* (p = 0.002), which was more favourable in football players (GG 98.5%) than elite endurance athletes (88.80%), and the *GSTP* (p = 0.014) and *GSTT* genotypes (p<0.049), which presented a more favourable genetic score in elite endurance athletes than football players (56.9% vs. 42.4% and 45.0% vs. 38.6% respectively). Differences between endurance athletes and non-athletes were found only in the *CYP2D6* polymorphism (p < 0.001), while in the professional football players and non-athlete population they were found in the *CYPD2D6* (p < 0.001) and *GSTT* (p = 0.003) genes (Table 1).

**Table 1:** Genotype distribution in professional athletes, elite endurance athletes, professional football players and non-athletes of liver metabolism polymorphisms.

Symbol	Gene	Polymorphism	dbSNP	Genotype Score	Professional athletes	Elite endurance athletes	Professional football players	Elite Endurance athletes vs. Professional football players p-value	non-athletes	Professional athletes vs. non-athletes p-value	Elite endurance athletes vs. non-athletes p-value	Professional football players vs. non-athletes p-value
***CYP2D6***	cytochrome P450 family 2 subfamily D member 6	c.506-1G>A	rs3892097	2 = GG	272 (93.2%)	142 (88.8%)	130 (98.5%)	0.002	98 (61.1%)	< 0.001	< 0.001	<0.001
				1 = GA	18 (6.2%)	17 (10.6%)	1 (0.8%)		59 (37.0%)			
				0 = AA	2 (0.7%)	1 (0.6%)	1 (0.8%)		3 (1.9%)			
***GSTM1***	glutathione-S transferase mu isoform 1	"Null" polymorphism		1 = +	121 (41.4%)	59 (36.9%)	62 (47.0%)	0.081	57 (35.7%)	0.389	0.999	0.104
				0 = -	171 (58.6%)	101 (63.1%)	70 (53.0%)		103 (64.3%)			
***GSTP***	glutathione S-transferase pi	c.313A>G	rs1695	2 = AA	147 (50.3%)	91 (56.9%)	56 (42.4%)	0.014	80 (50.0%)	0.975	0.306	0.423
				1 = GA	116 (39.7%)	59 (36.9%)	57 (43.2%)		64 (39.9%)			
				0 = GG	29 (10.0%)	10 (6.3%)	19 (14.4%)		16 (10.1%)			
***GSTT***	glutathione S-transferase theta	+/-		2 = +/+	123 (42.1%)	72 (45.0%)	51 (38.6%)	<0.001	64 (40.0%)	0.205	0.779	0.003
				1 = +/-	89 (30.5%)	34 (21.3%)	55 (41.7%)		39 (24.4%)			
				0 = -/-	80 (27.4%)	54 (33.8%)	26 (19.7%)		57 (35.6%)			

### Polygenic profile of iron metabolism and energy efficiency

When adding the genotype scores of *HFE*, *AMPD1* and *PGC1a* polymorphisms, the mean value of the TGS in professional athletes was 49.7 a.u. (±12.0 a.u.), statistical kurtosis: 0.13 (±0.28). For the group of elite endurance athletes, it was 51.1 a.u. (±11.6 a.u.), statistical kurtosis: 0.38 (±0.38) and in the professional football players, it was 48.1 a.u. (±12.4 a.u.), statistical kurtosis: -0.11 (±0.41). The mean value of the TGS in the non-athletes was 43.3 a.u. (±11.9 a.u.) statistical kurtosis: -0.46 (±0.43). The TGS values of the professional athletes and non-athletes were statistically significant (p < 0.001). Similar between elite endurance athletes and professional football players with non-athletes (p = 0.004 and p = 0.002 respectively).

**Fig 3:**
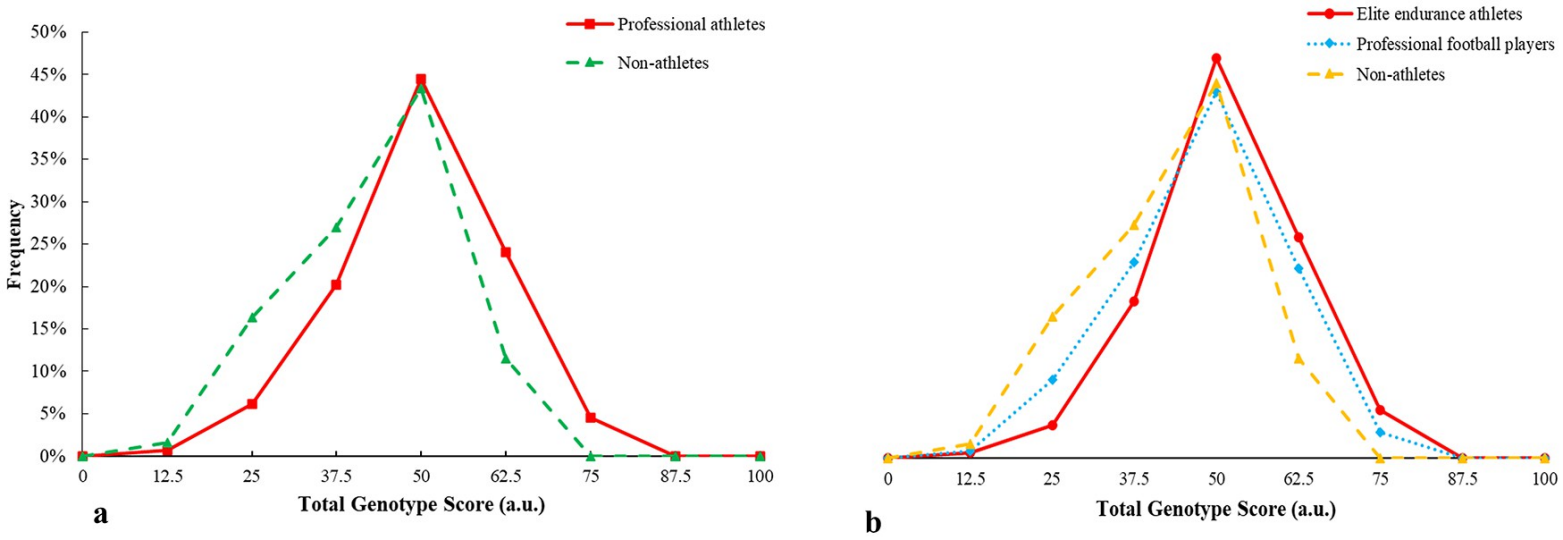
TGS distribution of iron metabolism and energy efficiency in a) professional athletes and non-athlete subjects and b) elite endurance athletes and professional football players with regard to non-athlete subjects.

TGS distribution of iron metabolism and energy efficiency genes in the professional athletes is shifted to the right with respect to non-athletes (p < 0.001) (Fig 3a), similar between professional football players (p = 0.044) and endurance athletes (p < 0.001) with respect to non-athletes (Fig 3b).

**Fig 4:**
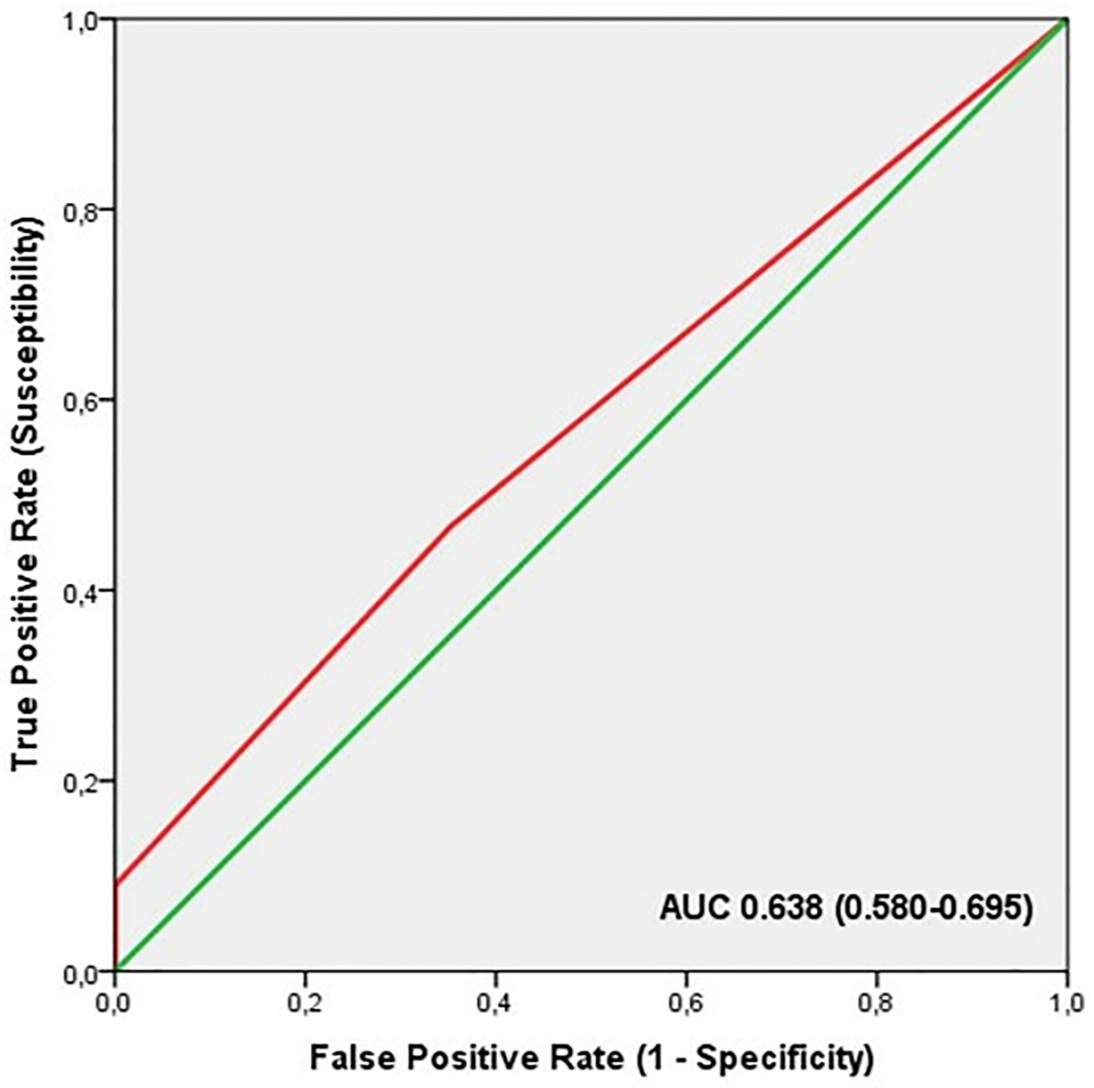
ROC curve summarising the ability of TGS of iron metabolism and energy efficiency genes to distinguish potential professional athletes from non-athletes.

ROC analysis showed significant discriminatory accuracy of TGS in the identification of professional athletes (AUC = 0.638; 95% CI: 0.580–0.695; p < 0.001) (sensitivity = 0.729, specificity = 0.549) (Fig 4). The corresponding TGS value at this point was 43.7 a.u. Binary logistic regression analysis showed that subjects with a higher TGS of 43.7 a.u. had an OR of 2.21 (95% CI: 1.42–3.43; p < 0.001) of being professional athletes, compared to those with a TGS below this value. The elite endurance athletes showed an OR at the cut-off point in comparison to the non-athlete population of 2.82 (95% CI: 1.69–4.73; p < 0.001) and professional football players, in comparison to non-athlete subjects, had an OR of 1.69 (95% CI: 1.02–2.82; p = 0.041).

Genotype distribution of iron metabolism and energy efficiency genes in the professional athlete’s group, when compared with the non-athlete population, was statistically significant for *HFE* c.187C>G (p = 0.001), showing a higher frequency in the “optimal” genotype in athletes (GG 5.8%) than the non-athlete population (GG 0.0%) and *AMPD1* CC genotype (94.2% vs. 62.1% respectively; p = 0.006) (Table 2). Between both groups of professional athletes (endurance and football players), statistically significant results were found in *HFE* c.187C>G showing genotypes more favourable in iron absorption in endurance athletes than in professional football players (p = 0.001). Differences between endurance athletes and the non-athlete population was found in the *HFE* c.187C>G polymorphism (p < 0.001), similar in professional football players and the non-athlete population (p = 0.013), presenting similar results in the *AMPD1* polymorphism between endurance athletes and non-athletes (p = 0.010) and professional football players and the non-athlete population (p = 0.014) (Table 2).

**Table 2:** Genotype distribution in professional athletes, elite endurance athletes, professional football players and non-athletes of iron metabolism and energy efficiency polymorphisms.

Symbol	Gene	Polymorphism	dbSNP	Genotype Score	Professional athletes	Elite endurance athletes	Professional football players	Elite endurance athletes vs. Professional football players p-value	non-athletes	Professional athletes vs. non-athletes p-value	Elite endurance athletes vs. non-athletes p-value	Professional football players vs. non-athletes p-value
***HFE***	Homeostatic Iron Regulator	c.187C>G	rs1799945	2 = GG	16 (5.8%)	8 (5.0%)	8 (6.1%)	0.001	0 (0.0%)	0.001	<0.001	0.013
				1 = GC	115 (39.0%)	78 (48.8%)	37 (28.0%)		43 (26.8%)			
				0 = CC	161 (55.2%)	74 (46.2%)	87 (65.9%)		117 (73.2%)			
***HFE***	Homeostatic Iron Regulator	c.845G>A	rs1800562	2 = AA	1 (0.3%)	0 (0.0%)	1 (0.8%)	0.449	0 (0.0%)	0.621	0.998	0.404
				1 = GA	16 (5.5%)	10 (6.3%)	6 (4.5%)		10 (6.3%)			
				0 = GG	275 (94.2%)	150 (93.7%)	125 (94.7%)		150 (93.7%)			
***AMPD1***	Adenosine monophosphate deaminase 1	c.34C>T	rs17602729	2 = CC	233 (79.8%)	128 (80.0%)	105 (79.6%)	0.291	99 (62.1%)	0.006	0.010	0.014
				1 = CT	57 (19.5%)	32 (20.0%)	25 (18.9%)		60 (37.4%)			
				0 = TT	2 (0.7%)	0 (0.0%)	2 (1.5%)		1 (0.5%)			
***PGC1a***	Peroxisome proliferator activated receptor coactivator	c.1444 G>A	rs8192678	2 = GG	192 (65.7%)	105 (65.6%)	87 (65.9%)	0.347	88 (54.8%)	0.232	0.523	0.119
				1 = GA	91 (31.1%)	53 (33.1%)	38 (28.8%)		63 (39.7%)			
				0 = AA	9 (3.2%)	2 (1.3%)	7 (5.3%)		9 (5.5%)			

### Polygenic profile of cardiorespiratory fitness

When adding the genotype scores of *ACE*, *NOS3*, *ADRA2A*, *ADRB2* and *BDKRB2* polymorphisms, the mean value of the TGS in professional athletes had a value of 53.6 a.u. (±13.0 a.u.), statistical kurtosis: 0.06 (±0.28). That of the group of elite endurance athletes was 51.1 a.u. (±11.6 a.u.), statistical kurtosis: 0.14 (±0.38) and in professional football players it was 54.8 a.u. (±13.6 a.u.), statistical kurtosis: -0.13 (±0.42). The mean value of the TGS in non-athletes was 51.7 a.u. (±12.0 a.u.) statistical kurtosis: -0.18 (±0.43). The TGS values of non-athletes and professional athletes were not statistically significant but there were differences between professional soccer players and the non-athlete population (p = 0.041).

**Fig 5:**
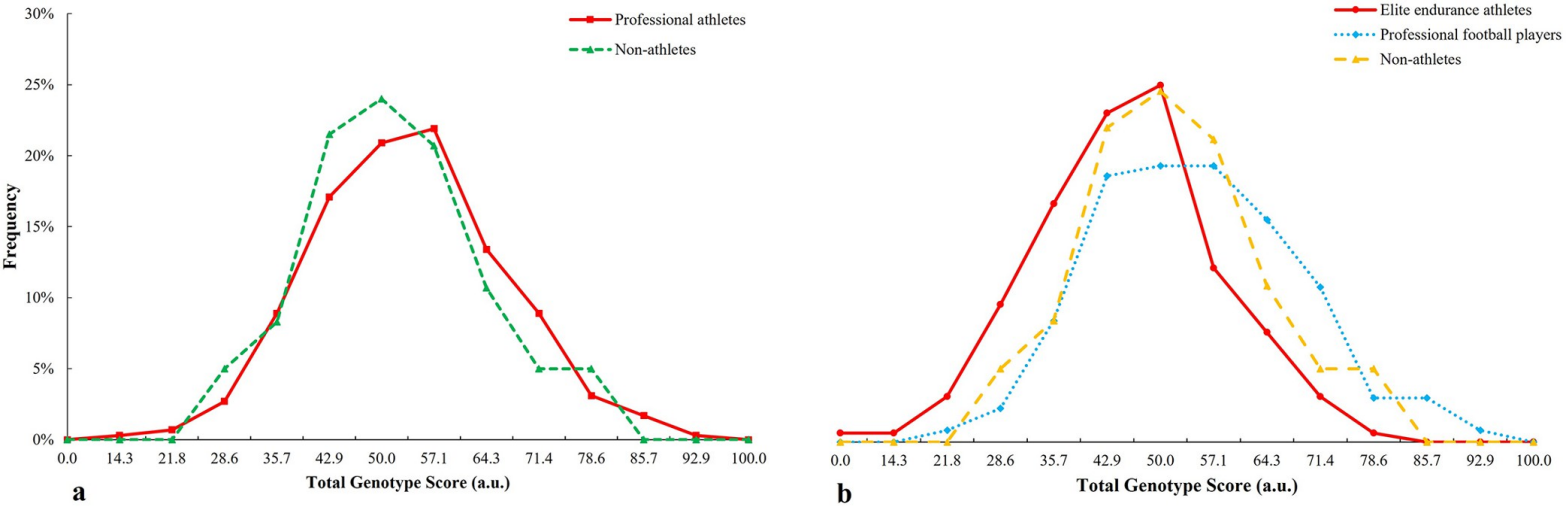
TGS distribution of cardiorespiratory fitness in a) professional athletes and non-athlete subjects and b) endurance athletes and football players with regard to non-athlete subjects.

TGS distribution of cardiorespiratory fitness genes in professional athletes was similar with respect to non-athletes (p = 0.590) (Fig 5a), similar between professional football players and endurance athletes (p = 0.282) with respect to non-athletes (p = 0.830) (Fig 5b).

**Fig 6:**
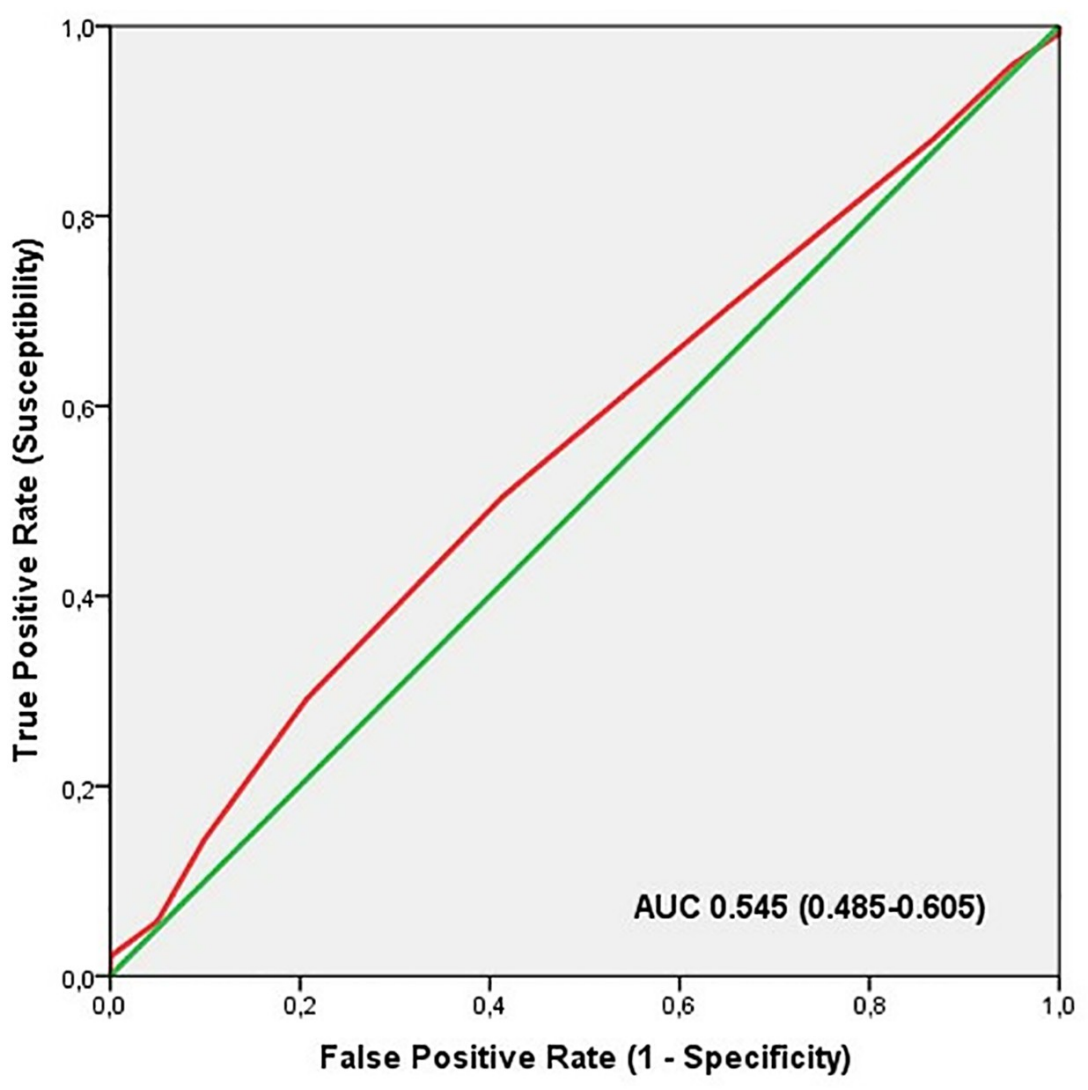
ROC curve summarising the ability of TGS in cardiorespiratory fitness genes to distinguish potential professional athletes from non-athletes.

ROC analysis in this profile did not show significant discriminatory accuracy of TGS in the identification of professional athletes (AUC = 0.545; 95% CI: 0.485–0.605; p = 0.152) (sensitivity = 0.493, specificity = 0.413) (Fig 6). The corresponding TGS value at this point was 53.5 a.u. Binary logistic regression analysis showed that subjects with a higher TGS of 53.5 a.u. had an OR of 1.38 (95% CI: 0.90–2.12; p = 0.129) of being professional athletes, compared to those with a TGS below this value. The elite endurance athletes showed an OR at the cut-off point in comparison to the non-athlete population of 1.28 (95% CI: 0.79–2.06; p = 0.303) and professional football players, in comparison to non-athlete subjects, had an OR of 1.50 (95% CI: 0.91–2.48; p = 0.105).

Genotype distribution of cardiorespiratory fitness genes in the professional athletes’ group when compared with the non-athlete population was statistically significant for *ACE* (p = 0.006), showing a higher frequency in the “non-optimal” genotype in professional athletes (DD 47.9%) than the non-athlete population (DD 38.7%), and similar in the *ADRA2A* c.-1291C>G GG genotype (11.5% vs. 3.3% respectively; p = 0.010). However, in the *ADRB2* c.79C>G polymorphism, the professional athletes showed a higher frequency in the “optimal” genotype (CC 31.5%) than the non-athlete population (16.00%) (p < 0.001) (Table 5). Between both groups of professional athletes (endurance and football players), statistically significant results were found in *NOS3* c.-786T>C showing a genotype more favourable in endurance athletes than in professional football players (p = 0.037), However, in the polymorphisms *ADRB2* c.46A>G and *BDKRB2*–9/+9 more favourable genotypes were found in professional football players than in endurance athletes (p = 0.034 and p < 0.001 respectively). Differences between endurance athletes and non-athletes were found in the *ACE* I/D polymorphism (p = 0.011), *NOS3* c.-786T>C (p = 0.005), *BDKRB2*–9/+9 (p = 0.003). Statistical differences were found in the *ADRA2A* c.-1291C>G and *ADRB2* c.79C>G polymorphisms in professional football players regarding the non-athlete population (Table 3).

**Table 3:** Genotype distribution in professional athletes, elite endurance athletes, professional football players and non-athletes of cardiorespiratory fitness polymorphisms.

Symbol	Gene	Polymorphism	dbSNP	Genotype Score	Professional athletes	Elite endurance athletes	Professional football players	Elite endurance athletes vs. Professional football players p-value	non-athletes	Professional athletes vs. non-athletes p-value	Elite endurance athletes vs. non-athletes p-value	Professional football players vs. non-athletes p-value
***ACE***	Angiotensin I-converting enzyme	Alu 287bp (I/D)	rs4340	2 = II	38 (13.0%)	25 (15.6%)	13 (9.8%)	0.175	16 (10.1%)	0.034	0.011	0.276
				1 = ID	114 (39.1%)	56 (35.0%)	58 (43.9%)		82 (51.2%)			
				0 = DD	140 (47.9%)	79 (49.4%)	61 (46.3%)		62 (38.7%)			
***NOS3***	Nitric Oxide Synthase 3	c.-786T>C	rs2070744	2 = TT	130 (44.6%)	82 (51.2%)	48 (36.4%)	0.037	48 (30.3%)	0.044	0.005	0.506
				1 = TC	114 (39.0%)	54 (33.8%)	60 (45.5%)		67 (41.8%)			
				0 = CC	48 (16.4%)	24 (15.0%)	24 (18.1%)		45 (27.9%)			
***NOS3***	Nitric Oxide Synthase 3	c.894G>T	rs1799983	2 = GG	137 (46.9%)	71 (44.4%)	66 (50.0%)	0.406	63 (39.1%)	0.227	0.329	0.201
				1 = GT	136 (46.6%)	80 (50.0%)	56 (42.4%)		81 (50.8%)			
				0 = TT	19 (6.5%)	9 (5.6%)	10 (7.6%)		16 (10.1%)			
***ADRA2A***	Adrenoceptor α-2a	c.-1291C>G	rs1800544	2 = CC	135 (46.2%)	71 (44.2%)	64 (48.5%)	0.759	90 (56.4%)	0.010	0.008	0.033
				1 = GC	123 (42.3%)	69 (43.5%)	54 (40.9%)		66 (41.3%)			
				0 = GG	34 (11.5%)	20 (12.3%)	14 (10.6%)		4 (2.3%)			
***ADRB2***	Adrenergic receptor β-2	c.46A>G	rs1042713	2 = AA	33 (11.3%)	15 (9.4%)	18 (13.6%)	0.034	14 (9.1%)	0.607	0.751	0.119
				1 = GA	146 (50.0%)	91 (56.9%)	55 (41.7%)		87 (54.4%)			
				0 = GG	113 (38.7%)	54 (33.7%)	59 (44.7%)		59 (36.5%)			
***ADRB2***	Adrenergic receptor β-2	c.79C>G	rs1042714	2 = CC	92 (31.5%)	43 (26.9%)	49 (37.1%)	0.056	26 (16.0%)	<0.001	<0.001	<0.001
				1 = GC	164 (56.2%)	100 (62.5%)	64 (48.5%)		77 (48.3%)			
				0 = GG	36 (12.3%)	17 (10.6%)	19 (14.4%)		57 (35.7%)			
***BDKRB2***	Bradykinin Receptor β2	+9 pb/-9 pb	rs5810761	2 = -9/-9	74 (25.3%)	33 (20.6%)	41 (31.1%)	<0.001	38 (23.8%)	0.153	0.003	0.373
				1 = -9/+9	129 (44.2%)	61 (38.1%)	68 (51.5%)		84 (52.4%)			
				0 = +9/+9	89 (30.5%)	66 (41.3%)	23 (17.4%)		38 (23.8%)			

### Polygenic profile for muscle injuries

When adding the genotype scores of *ACE*, *ACTN3*, *AMPD1*, *MLCK* and *CKM* polymorphisms, the mean value of the TGS in professional athletes had a value of 58.3 a.u. (±11.5 a.u.), statistical kurtosis: -0.20 (±0.28). That of the group of elite endurance athletes was 57.9 a.u. (±11.8 a.u.), statistical kurtosis: -0.29 (±0.38) and in professional football players was 58.7 a.u. (±11.1 a.u.), statistical kurtosis: -0.05 (±0.42). The mean value of the TGS in non-athletes was 51.2 a.u. (±10.8 a.u.) statistical kurtosis: 0.11 (±0.43). The TGS values of the professional athletes and non-athletes were statistically significant (p < 0.001). Similar between elite endurance athletes and professional football players with non-athletes (all p < 0.001).

**Fig 7:**
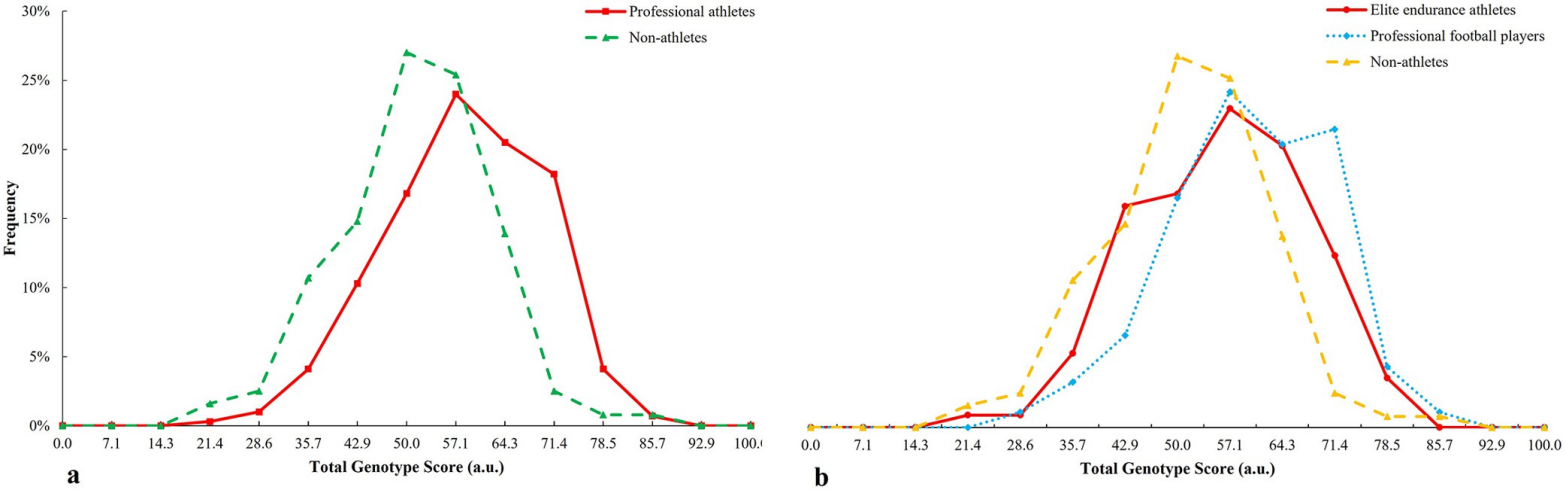
TGS distribution for muscle injuries genes in a) professional athletes and non-athlete subjects and b) endurance athletes and football players with regard to non-athlete subjects.

TGS distribution for muscle injuries genes in professional athletes is shifted to the right with respect to non-athletes (p < 0.001) (Fig 7a), showing similar results between elite endurance athletes and professional football players with non-athletes (p < 0.001) (Fig 7b).

**Fig 8:**
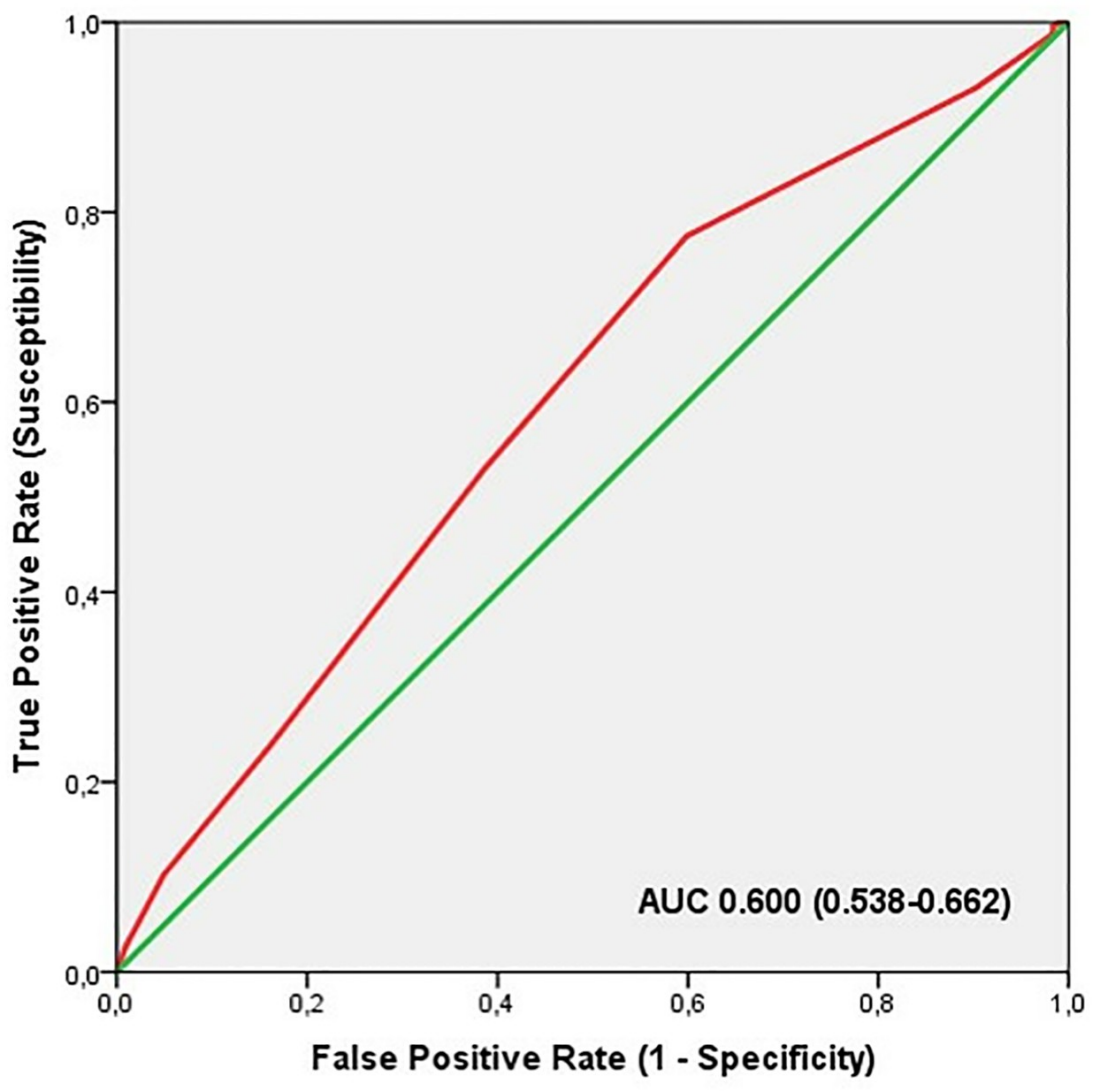
ROC curve summarising the ability of TGS for muscle injuries genes to distinguish potential professional athletes from non-athletes.

ROC analysis in this profile did not show significant discriminatory accuracy of the TGS in the identification of professional athletes (AUC = 0.600; 95% CI: 0.538–0.662; p = 0.002) (sensitivity = 0.775, specificity = 0.598) (Fig 8). The corresponding TGS value at this point was 53.5 a.u. Binary logistic regression analysis showed that subjects with a lov TGS of 53.5 a.u. had an OR of 2.70 (95% CI: 1.75–4.16; p < 0.001) of being professional athletes, compared to those with a TGS below this value. The elite endurance athletes showed an OR at the cut-off point in comparison to the non-athlete population of 2.48 (95% CI: 1.53–4.03; p < 0.001) and professional football players, in comparison to non-athlete subjects, had an OR of 2.99 (95% CI: 1.78–5.01; p < 0.001).

Genotype distribution for muscle injuries polymorphisms in the professional athletes’ group when compared with the non-athlete population was statistically significant for *ACE* I/D (p = 0.034), showing a higher frequency in the “protective” genotype in athletes (DD 47.9%) than the non-athlete population (DD 38.7%), *AMPD1* CC genotype (79.8% vs. 62.1% respectively; p = 0.006) and “protective” c.37885C>A and c.49C>T *MLCK* polymorphisms (p < 0.001) (Table 4). Between both groups of professional athletes (endurance and football players), statistically significant results were found in *MLCK* polymorphisms showing genotypes more protective in professional football players than in endurance athletes (c.37885C>A; p = 0.002 and c.49C>T; p = 0.001). Differences between endurance athletes and non-athletes were found in the *ACE* polymorphism (p = 0.001), presenting similar results in the *AMPD1* polymorphism between endurance athletes, and the non-athlete population (p = 0.013) and professional football players and non-athletes (p = 0.014). In *MLCK* c.37885C>A and c.49C>T polymorphisms statistically significant differences were found between both groups of endurance athletes and professional football players and the non-athlete population (p < 0.001) (Table 4).

**Table 4:** Genotype distribution in professional athletes, elite endurance athletes, professional football players and non-athletes for muscle injuries polymorphisms.

Symbol	Gene	Polymorphism	dbSNP	Genotype Score	Professional athletes	Elite endurance athletes	Professional football players	Elite endurance athletes vs. Professional football players p-value	non-athletes	Professional athletes vs. non-athletes p-value	Elite endurance athletes vs. non-athletes p-value	Professional football players vs. non-athletes p-value
***ACE***	Angiotensin I-converting enzyme	Alu 287bp (I/D)	rs4340	2 = DD	140 (47.9%)	79 (49.4%)	61 (46.2%)	0.175	62 (38.7%)	0.034	0.011	0.216
				1 = ID	114 (39.0%)	56 (35.0%)	58 (43.9%)		82 (51.2%)			
				0 = II	38 (13.1%)	25 (15.6%)	13 (9.9%)		16 (10.1%)			
***ACTN3***	α-actinin 3	c.1729C>T	rs1815739	2 = CC	91 (31.2%)	55 (34.4%)	36 (27.3%)	0.211	48 (30.0%)	0.681	0.678	0.410
				1 = TC	150 (51.4%)	82 (51.2%)	68 (51.5%)		91 (56.6%)			
				0 = TT	51 (17.4%)	23 (14.4%)	28 (21.2%)		21 (13.4%)			
***AMPD1***	Adenosine Monophosphate Deaminase 1	c.34C>T	rs17602729	2 = CC	233 (79.8%)	128 (80.0%)	105 (79.6%)	0.291	99 (62.1%)	0.006	0.010	0.014
				1 = CT	57 (19.5%)	32 (20.0%)	25 (18.9%)		60 (37.4%)			
				0 = TT	2 (0.7%)	0 (0.0%)	2 (1.5%)		1 (0.5%)			
***CKM***	Muscle-specific creatine kinase	c.*800A>G	rs8111989	2 = GG	26 (8.9%)	16 (10.0%)	10 (7.6%)	0.418	10 (5.9%)	0.718	0.326	0.384
				1 = GA	142 (48.6%)	88 (55.0%)	54 (40.9%)		72 (45.3%)			
				0 = AA	124 (42.5%)	56 (35.0%)	68 (51.5%)		78 (48.8%)			
***MLCK***	Myosin-light chain kinase	c.37885C>A	rs28497577	2 = AA	1 (0.4%)	0 (0.0%)	1 (0.8%)	0.002	1 (0.6%)	<0.001	<0.001	<0.001
				1 = CA	217 (74.3%)	107 (66.9%)	110 (83.3%)		56 (35.3%)			
				0 = CC	74 (25.3%)	53 (33.1%)	21 (15.9%)		103 (64.1%)			
***MLCK***	Myosin-light chain kinase	c.49C>T	rs2700352	2 = CC	167 (57.2%)	79 (49.4%)	88 (66.7%)	0.001	31 (19.6%)	<0.001	<0.001	<0.001
				1 = CT	99 (33.9%)	59 (36.8%)	40 (30.3%)		109 (68.1%)			
				0 = TT	26 (8.9%)	22 (13.8%)	4 (3.0%)		20 (12.3%)			

All genetic profiles, through binary logistic regression showed different prediction values of being professional athletes, both endurance athletes and professional football players, in the genes presented, with reference to non-athletes, as shown in Table 5.

**Table 5:** Prediction values of polygenic profiles of being professional athletes, elite endurance athletes and professional football players.

Polygenic Profiles	Professional athletes		Elite endurance Athletes		Professional football players	
	OR^a^ (95% CI)^b^	p-value	OR^a^ (95% CI)^b^	p-value	OR^a^ (95% CI)^b^	p-value
***Liver metabolism***	1.96 (1.28–3.01)	0.002	1.79 (1.11–2.88)	0.017	2.20 (1.32–3.66)	0.001
***Iron metabolism and energy efficiency***	2.21 (1.42–3.43)	<0.001	2.82 (1.69–4.73)	<0.001	1.67 (1.02–2.82)	0.041
***Cardiorespiratory fitness***	1.38 (0.90–2.12)	0.129	1.28 (0.79–2.07)	0.303	1.51 (0.91–2.48)	0.105
***Muscle Injuries***	2.70 (1.75–4.16)	<0.001	2.48 (1.53–4.03)	<0.001	2.99 (1.78–5.***01***)	<0.001

^a^OR: Odds Ratio

^b^95% CI: 95% Confidence interval

## Discussion

Previous research has been satisfactory in finding links between potential genetic markers associated with enhanced physiological functioning and professional sports performance [8, 59, 60]. Although polygenic profiles of athletes have previously been identified, this is the first study to investigate the polygenic profiles of several distinct physiological systems in elite endurance athletes, professional football players and the non-athlete population in a sample of homogeneously selected individuals.

It is known that in competitions like cycling and elite running the accumulated efforts over several weeks affect performance, which is also the case in professional football, due to the alteration in the redox-system of the systemic homeostasis and withdrawal of toxic products generated by the high oxidative stress produced by these sports disciplines [61, 62]. This oxidative stress in professional athletes is a determinant of performance. In this respect, the comparisons presented in the polygenic profile of liver metabolism, previously defined by Varillas et al. [24], in which it showed that endurance athletes had a higher systemic recovery capacity than the non-athlete population, presenting a metabolism that scavenges free radicals and oxygen peroxides produced by high-performance sports, are further demonstrated in this research. We propose that professional football players also present optimal hepatic metabolism genetics with respect to the non-athlete population, even with an OR higher than elite endurance athletes, thus indicating that the contact and strength exercise adapts this cohort of football players to more effective systemic cleaning (Table 5).

By using a polygenic model, it has been shown for the first time that polymorphic variations in iron metabolism and energy efficiency genes had a joint effect on the probability of becoming a professional athlete, as previously shown in a recent article [5], adding this cohort of professional football players. The significant “favourability” in the genetic profile studied in professional athletes, elite endurance athletes and professional football players versus non-athletes, presenting in elite endurance athletes a favourable adaptative factor, especially conditioned by the c.187C>G polymorphism of the *HFE* gene, as well as the c.34C>T polymorphism of the *AMPD1* gene in both groups of athletes (Table 4), has been shown in a previous study by Ruiz et al. [33]. in genes predictive of endurance with 7 different markers associated with sports performance.

Previous studies have shown the association of the c.187C>G polymorphism of the *HFE* gene with professional athletes [22, 35, 63], as well as the involvement of the *AMPD1* polymorphism in early muscle fatigue [64–66], revealed for the first time in professional football players, presenting allelic frequencies in these genes similar to elite endurance athletes, indicating that professional football players could present genetic factor conditioning that makes them resemble elite endurance athletes in these polygenic profiles, both in iron metabolism, energy efficiency and liver metabolism.

Cardiorespiratory fitness refers to the capacity of the circulatory and respiratory systems to supply oxygen to skeletal muscle mitochondria for energy production needed during physical activity [67, 68]. Cardiorespiratory fitness is positively associated with power and endurance exercise performance [62] and is a strong prognostic factor of morbidity and mortality from all causes and, particularly, from cardiovascular disease (CVD) [69]. The measure of an individual’s peak capacity to perform dynamic aerobic exercise is dependent on the synergistic action of pulmonary, cardiovascular and muscle tissue via a suite of physiological actions that effectively transport and deliver oxygen from the atmosphere to the mitochondria in working muscles [36, 37].

Although these interindividual variations have been previously described, the polygenic profile in polymorphisms of *ACE*, *NOS3*, *ADRA2A*, *ADRB2* and *BDKRB2* genes was similar in elite endurance athletes and in the non-athlete population, suggesting that the combination of these genes does not determine endurance performance. However, differences in TGS in cardiovascular aptitude were only shown between professional football players and the non-athlete population, suggesting that these genetic markers could be selective for successful professional football practice due to more extreme cardiovascular work than that which occurs in endurance sports, showing for the first time an “optimal” genetic profile in cardiorespiratory fitness which differentiates professional football players, from endurance athletes and the non-athlete population.

The most important finding of this study was the “favourability” of the polygenic profile in muscle injuries to professional athlete status in elite endurance athletes and professional football players.

Although excellent muscle performance in endurance sports is facilitated by an optimal polygenic profile in several key polymorphisms by muscle fibres, elasticity and metabolism [5, 24, 70, 71], this analysis indicates that the influence of these five genes is strong enough to discriminate this profile and the risk of muscle injuries [40]. The most protectives polymorphisms for muscle injuries in professional athletes compared to the non-athlete population were those in the *ACE*, *AMPD1* and *MLCK* (Table 4). The c.1729C>T polymorphism of *ACTN3* gene widely studied and correlated with sports performance [12, 47, 72] in the largest cohort of professional athletes presented in the literature to date, which shows no association and relevance in the identification of sports talents due to its lack of relationship to muscle injuries and not affecting sports performance [20, 59], an aspect that should be expanded in the relationship of the polymorphism of *ACTN3* c.1729C> T with sports injuries in subsequent studies.

In turn, there were differences between both groups of professional athletes and the non-athlete population in the *AMPD1* and *MLCK* genes, these being the genes that most predict “optimal” metabolism, strength and possible prevention of sports injuries (Table 4). Between the two groups of professional athletes, the polymorphisms c.37885C>A and c.49C>T of the *MLCK* gene present more “optimal” genetic values in professional football players than in elite endurance athletes, being protective predictors of loss of muscle strength after exercise that is produced more in football matches compared to endurance competitions [53, 54], favouring greater muscular force in football players in comparison to elite endurance athletes, presenting for the first time this genetic adaptation between these sport modalities in the largest and most homogeneous cohort analysed, being "protective" in the risk of suffering muscular injuries, an aspect that increases the capacity for sports performance and thus favours the status of elite athlete (Table 4).

Whilst genetic testing has the potential to assist in the identification of future talented performers, genetic tests should be combined with other tools, such as physiological values, and injury data to obtain an accurate identification of those athletes predisposed to succeed in the sport. Previous studies show the importance of the use of total genotype scores, composed of a high number of performance-enhancing polymorphisms, which will likely be one of the best strategies in the utilisation of genetic information to identify talent in sport [11], as presented in this research. The genetic information may represent a potentially useful adjunct to existing talent identification procedures, enhancing the process of selection. Such information should not be used as a standalone, but as an adjunct to current talent identification processes, thereby allowing the training process to become more personalised, and enabling athletes to get ever closer to their maximum potential.

Currently, we cannot use genetic information for the identification of talent because few genes have been discovered related to sports performance and many of the results shown in the literature are still contradictory [9, 10]. These contradictory effects demonstrate that there is not a singular genetic profile that confers sporting success, but that the required genetic profiles are likely specific for the characteristics of each sport [9, 11].

Accordingly, at present, only a few of the genetic markers are known that likely associate with elite athlete status, making predictions of future sporting performance based on such information not only difficult but also probably inaccurate [9, 10, 73]. In addition, testing the utility and efficacy of genetic testing in the talent identification process should be conducted to ascertain whether the information provided by genetic testing and not obtained through other traditional non-genetic tests such as physical testing, is of relevance to increase the specificity of overall talent selection [73].

For the first time, to the best of our knowledge, the relationship between these polymorphisms involved in liver metabolism, iron metabolism and energy efficiency, cardiorespiratory fitness and muscle injuries target genes is shown, leading to defining the capacity to become a professional athlete. This is a new type of genetic study, showing a definitive model of the polygenic profiles that help the capacity of physical effort in this group of subjects contributing to understanding the multiple and complex mechanisms that define it.

Subsequent studies should be carried out that amplify these polygenic profiles in elite endurance athletes, professional football players and elite athletes in sprint events to determine their ability to reach a high sports performance to corroborate the results shown in this study and to be able to conclude that these genetic markers are predisposed to support optimal talent identification in high sports performance, in order to discriminate the good from the best athletes.

Our findings demonstrates that the genetic distribution in professional athletes as regards endurance (professional cyclist and elite runners) and professional football players is different to the non-athlete population, there being a favourable polygenic profile in terms of liver metabolism, iron metabolism and energy efficiency and muscle injuries.

These genetic data presented among the analysed professional athletes show a concordance between elite endurance athletes and professional football players, supporting for the first time that players of this sports modality present genetic characteristics similar to endurance sports athletes.

These results open up a new path of research into these gene groups to complete knowledge on talent identification for high sports performance in professional athletes.

## Materials and methods

### Study design

A transversal prospective study.

### Subjects

Four hundred and fifty-two subjects were recruited: 160 elite male endurance athletes (112 professional cyclists from Union Cycliste Internationale (UCI) World Tour teams, competing in grand tours, being UCI world champions and Grand Tour winners, with victories in the UCI World Tour and Continental Pro Tour races; and 48 elite long-distance runners: 5,000 m to marathon athletes who have competed in top-level races at the Olympic Games, World Championships, and European Championships in the marathon, half-marathon, and cross country); 132 male professional football players (from the Spanish Liga Santander and Liga Smartbank with several of them competing in the Union of European Football Associations (UEFA) Champions League and UEFA Europa League competitions representing their respective countries in international events), and 160 non-athlete male subjects. The non-athletes were matched by age with the elite athletes, the inclusion criteria being that they be non-smokers, and not suffering from chronic or acute diseases, or obesity, at the time of sampling.

Participation and acceptance of inclusion of participants were obtained by signing the informed consent document. The study protocol was approved by the Ethics Committee of the Universidad Francisco de Vitoria (32/2020) and was in accordance with the Declaration of Helsinki for Research in Humans of 1964 (last modified in 2013).

### Genotypes

#### Target genes

In order to investigate the role of different genetic variants related to the status of elite athletes, the following polygenic profiles were selected with functional polymorphisms in the following target genes.

#### Liver metabolism

The polymorphisms studied were previously presented as *CYP2D6* (rs3892097), *GSTM1*, *GSTP* (rs1695) and *GSTT*, and have been previously associated in relation to their influence on the detoxification capacity of elite athletes [24], having been presented previously as markers of drug metabolism in different pathologies [74–76].

#### Iron metabolism and energy efficiency

In this genetic profile, the polymorphisms of *HFE* c.187C>G (rs1799945) and c.845G>A (rs1800562), *AMPD1* c.34C>T (rs17602729) and *PGC1α* c.1444 G>A (rs8192678), show a capacity for efficient iron absorption and muscle metabolism for optimal muscle performance in professional sports [5, 64, 65].

#### Cardiorespiratory fitness

The polymorphisms of *ACE* I/D (rs4340), nitric oxide synthase (*NOS3*) c.-786T>C (rs2070744) and c.894G>T (rs1799983), α2a adrenoceptor (*ADRA2a*) c.-1291C>G (rs1800544), β-2 adrenoceptor (*ADRB2*) c.46A>G (rs1042713) and c.79C>G (rs1042714) and bradykinin receptor B2 (*BDKRB2* -9b/+9pb (rs5810761) have been shown to enhance cardiovascular training in sports, increasing VO_2_max, strength and sports performance [18, 39, 77–79].

#### Muscle injuries

Polymorphisms of the genes *ACE* I/D (rs4340), *ACTN3* c.1729C>T (rs1815739), *AMPD1* c.34C>T (rs17602729), *CKM* c.-800A>G (rs8111989) *MLCK*: c.37885C>A (rs28497577) and c.49C>T (rs2700352) have previously been linked to muscle injury incidence as well as muscle damage after competitive exercise [43, 54, 80].

### Sample collection and genotyping

The extraction of DNA was carried out by oral smear with SARSTED swabs, and was kept cold until its extraction in the laboratory.

The extraction of genomic DNA from the oral mucosa samples was carried out by automatic extraction in QIACube equipment (QIAGEN, Venlo, Netherlands), with a DNA concentration yield of 25-40ng/ml, which was kept in solution in a volume of 100 microliters at -20ºC until genotyped.

All the selected genes were genotyped by multiplex analysis by Polymerase Chain Reaction-Single Nucleotide Primer Extension (PCR-SNPE), a multiplex-PCR for amplification of targeted sequences, followed by single-base extension assay of probe-primers using the commercial SNaPshot Kit (Applied Biosystems, Foster City, CA) in the Real Time-PCR instrument QuantStudio5 (QS5) (Thermofisher, CA).

### Polygenic potential for detection of professional athlete status

The combined influence of each of the SNPs in the different profiles was calculated following the Williams and Folland procedure [81]. Typically, the candidate polymorphisms were bi-allelic, except for GSTM which only provided one allele. According to previous research in athletic performance, SNPs were scored as follows; a score of 2 points was given to an optimal genotype score (GS), 1 point was scored for heterozygotes and non-favourable homozygotes scored 0 points, except for the GSTM gene whose optimal GS had a value of 1.

For each polygenic profile, the scores obtained in each genetic polymorphism were added up for a perfect total genotype score that represents the optimal genotype, which was for the liver metabolic profile 7 arbitrary units (a.u.) (range 0–7 a.u.), profile energy and iron 8 a.u. (range 0–8 a.u.), cardiorespiratory fitness of 14 u.a. (range 0–14 a.u.) and muscle performance of 12 a.u. (range 0–12 a.u.). Finally, this value was transformed into a scale of 0–100 a.u. to facilitate interpretation, namely the Total Genotype Score (TGS), as follows.

Liver metabolism:

$$TGS=\left(GS_{CYP2D6}+GS_{GSTM1}+GS_{GSTP}+GS_{GSTT}\right)\times \left(100/7\right)$$ (1)

Iron metabolism and energy efficiency:

$$TGS=\left(GS_{AMPD1}+GS_{PGC1a}+GS_{HFEH63D}+GS_{HFEC282Y}\right)x\left(100/8\right)$$ (2)

Cardiorespiratory fitness:

$$TGS=\left(GS_{ACE}+GS_{NOS3-786}+GS_{NOS3E298D}+GS_{ADRA2A-1291}+GS_{ADRB2R16}+GS_{ADRB2Q27E}+GS_{BDKRB2}\right)x\left(100/14\right)$$ (3)

Muscle injuries:

$$TGS=\left(GS_{ACE}+GS_{ACTN3}+GS_{AMPD1}+GS_{CKM}+GS_{MLCKC37885A}+GS_{MLCKC49T}\right)x\left(100/12\right)$$ (4)

As indicated above, a TGS of 100 a.u. represents a "perfect" profile and a TGS of 0 a.u. would be the "worst" profile possible when all GS have a score of 0 a.u. [81]. Finally, the distribution was evaluated of all TGS among the different profiles of professional athletes, elite endurance athletes, professional football players and non-athletes.

### Statistical analysis

The statistical average and kurtosis were calculated using the Statistical Package for the Social Sciences (SPSS), v.21.0 for Windows (IBM Corp. Released 2012. IBM SPSS Statistics for Windows, Version 20.0. Armonk, NY: IBM Corp., USA).

The HWE was tested for each SNP using χ2 tests. The probability of having an “optimal” soccer genotype for one to four genetic profiles between soccer players and non-athletes was calculated using the χ2 test with a fixed α 0.05.

The genotypic frequencies of the polymorphisms in liver-metabolising, energy and iron metabolising, cardiorespiratory fitness and muscle injuries profiles were compared between soccer players and non-athletes, using a χ2 test with fixed α 0.05. The ability of TGS to correctly distinguish potential professional athletes from non-athletes (0 = professional athlete, 1 = non-athlete) was assessed using receiver operating characteristic (ROC) curves [82]. With that purpose, the area under the ROC curve (AUC) was calculated with confidence intervals of 95% (95% CI). Finally, a binary logistic regression model was used to study the relationship between TGS and the elite endurance athlete and professional football player status.
